# Real-Time Neuropsychological Testing Protocol for Left Temporal Brain Tumor Surgery: A Technical Note and Case Report

**DOI:** 10.3389/fnhum.2021.760569

**Published:** 2021-12-03

**Authors:** Barbara Tomasino, Ilaria Guarracino, Tamara Ius, Marta Maieron, Miran Skrap

**Affiliations:** ^1^Scientific Institute, IRCCS E. Medea, Dipartimento/Unità Operativa Pasian di Prato, Udine, Italy; ^2^SOC Neurochirurgia, Azienda Sanitaria Universitaria Friuli Centrale ASU FC, Udine, Italy; ^3^Fisica Sanitaria, Azienda Sanitaria Universitaria Friuli Centrale ASU FC, Udine, Italy

**Keywords:** awake surgery, brain mapping, glioma, neuropsychology, plasticity

## Abstract

**Background:** The risk of surgery in eloquent areas is related to neuropsychological dysfunctions. Maximizing the extent of resection increases the overall survival. The onco-functional balance is mandatory when surgery involves cognitive areas, and maximal information on the cognitive status of patients during awake surgery is needed. This can be achieved using direct cortical stimulation mapping and, in addition to this, a neuropsychological monitoring technique called real-time neuropsychological testing (RTNT). The RTNT includes testing protocols based on the area where the surgery is performed. We reported on tests used for left temporal lobe surgery and our RTNT decision tree.

**Case Report:** We reported our RTNT experience with a 25-year-old right-handed man with 13 years of schooling. He reported daily partial seizures. MRI revealed the presence of a low-grade glioma involving the temporo-insular cortex. The neuropsychological status presurgery which was within the normal range was combined with functional magnetic resonance imaging (fMRI) and diffusion tensor imaging (DTI) information. Awake surgery plus RTNT was performed. Direct electrical stimulation during object naming elicited a motor speech arrest. Resection was continuously accompanied by the RTNT. The RTNT provided enriched information to the surgeon. Performance never dropped. A slight decrement in accuracy emerged for pseudoword repetition, short-term memory and working memory, phonological processing, and verbal comprehension. Total resection was performed, and the histological examination confirmed the nature of the lesion. Immediate postsurgery performance was within the normal range as it was the fMRI and DTI assessment.

**Conclusion:** The RTNT provides essential information that can be used online, during surgery, for clinical aims to provide the surgeon with useful feedback on the cognitive status of patients.

## Introduction

Resections in eloquent areas entail the risk of altering the cognitive capacities of patients (Sanai and Berger, 2008). Awake surgery is performed to preserve neuropsychological functions (Ojemann et al., 1989; Ojemann, 1993; Duffau et al., 2003, 2005).

Assessment of cognitive functioning is highly recommended as the neuropsychological profile is an important outcome (Ng et al., 2020). Neuropsychological deficits could arise because of tumor growth or surgery and may affect the quality of life. For instance, Ng et al. (2020) compared pre- and postsurgery cognitive assessments in a consecutive series of 47 patients with (incidental) low-grade glioma (LGG) who underwent awake surgery. Of note, 72.3% of the patients presented stable cognitive functioning, 14.9% of the patients improved cognitive functioning, and 12.8% of the patients showed slight impairments.

Sparing neuropsychological functions during awake surgery is crucial. However, only language or motor functions are routinely mapped. A review on the neuropsychological tests used during awake brain surgery (Ruis, 2018) reported that cognitive functions (i.e., memory, calculation, and emotions) were assessed in a minority of cases.

To test the neuropsychological functions during awake surgery, direct electrical stimulation (DES) mapping is performed (Berger and Ojemann, 1992; Berger, 1994, 1996; Duffau et al., 2000, 2005), while the patient carries out a cognitive task. DES mapping minimizes the risk of permanent postoperative deficits while maximizing the quality of resection, especially in infiltrating tumors such as LGGs where the larger the resection, the longer the survival of a patient (Duffau et al., 2003; Keles et al., 2004; Sanai and Berger, 2008, 2011; Sanai et al., 2010; Ius et al., 2012). The surgeon determines where DES evokes an interference response and imports this spatial information into a 3D MRI image to reproduce a somewhat personalized non-resectability map to guide surgery at cortical and subcortical levels. DES mapping is repeated intra-resections at intervals by pausing excision.

The most frequently used task for surgery in the left Sylvian and peri-Sylvian areas is naming (or counting; Duffau et al., 2000, 2002, 2003; Brennan et al., 2007; Tonn, 2007; Chang et al., 2017). DES mapping at cortical or subcortical portions of the brain induces an interference with task performance. For example, if the patient is carrying out a counting task, DES in Broca’s area may elicit speech arrest. As another example, during a naming task, DES mapping delivered in temporal areas may trigger anomia or phonological and semantic paraphasia. Sometimes, semantic processing can be tested by means of the Pyramids and Palm Trees Test (Talacchi et al., 2013).

Other tests for the left temporal lobe include repetition (Moritz-Gasser and Duffau, 2013; Sierpowska et al., 2017; Leonard et al., 2019). DES to the left peri-Sylvian network causes interference with word repetition with phonological errors, perceptual deficits, and speech arrest (Leonard et al., 2019). Dissociations between naming and pseudoword repetition can be observed: DES to the arcuate fasciculus causes interference with pseudoword repetition (Sierpowska et al., 2017), while DES to the posterior part of the superior and middle temporal gyri induces anomias with preserved word repetition (Moritz-Gasser and Duffau, 2013). These data suggest that it is worth using a multiple testing approach with more than one test.

There are occasional reports of DES during a reading task (Roux et al., 2004, 2012; Gil-Robles et al., 2013; Motomura et al., 2014; Zemmoura et al., 2015; Hirshorn et al., 2016). A study used a somewhat different approach and administered the reading test during resection and not during DES (Tomasino et al., 2020). A decrease in performance (vs. presurgery data) was found in 19/49 patients, and their *x*, *y*, and *z* brain coordinates, where resection causes a decrement, were distributed on the posterior inferior and middle temporal gyrus to the temporoparietal areas and the precentral gyrus (Tomasino et al., 2020).

There are reports on DES during writing (Roux et al., 2003, 2009, 2014; Lubrano et al., 2004; Motomura et al., 2014) and less data on a picture description task to test syntax (Chang et al., 2018).

In addition to DES mapping, surgeons may obtain further information from neuropsychological monitoring providing feedback on the cognitive status of patients during resection. This (Skrap et al., 2016) can be performed using real-time neuropsychological testing (RTNT). The RTNT is listed among the neuropsychological techniques (Tomasino and Rumiati, 2020) available for neuropsychologists as clinical tools. Contrary to DES, the RTNT is performed without pausing excision, as it is administered from the beginning of resection until hemostasis. Upon the request of a surgeon, the RTNT can be temporarily paused to perform DES. An RTNT protocol consists of a battery of neuropsychological tasks tapping the area under resection, e.g., for insular lobe, see Tomasino et al. (2018). It is individually selected for each patient based on the lesion localization, presurgical functional magnetic resonance imaging (fMRI)/diffusion tensor imaging (DTI) results, and presurgical neuropsychological status. Each RTNT task includes 10–15 items (e.g., pictures, sentences, words, and numbers to be repeated) and is repeated several times, by changing items, in order to test several cognitive functions in 1–2 min. Thus, the RTNT can provide continuous feedback on cognitive functions in real time in order to monitor the consequence of each surgical act. A decrease in performance might warrant pausing resection, after searching for confirmation of the functionality of the anatomical site using DES. If RTNT runs are performed correctly, the surgeon resumes resection. Finally, a temporary decrease in performance, which could be related to mechanical manipulation or fatigue of a patient, deserves in-depth investigation. During this time, a “stop-and-go” approach (Sala et al., 2007) is used by the surgeon.

In this case report, we presented our RTNT protocol for resections in the left temporal lobe and describe our multimodal approach combining neuropsychology, fMRI, DTI, and RTNT plus DES. By using the RTNT approach, we aimed to obtain a complete overview of the neuropsychological functions of a patient supported by the craniotomy site and to determine whether—and if so, how—the neuropsychological status evolves during resection. Second, we expected to confirm that this method can be applied in a short resection time.

## Materials and Methods

### Procedure

We first performed the presurgical neuropsychological assessment followed by fMRI and DTI examinations presurgery. The patient underwent awake surgery plus RTNT, immediate postsurgery neuropsychological assessment 1 week after surgery, and, after 4 months, neuropsychological testing and fMRI follow-up.

#### Real-Time Neuropsychological Testing Decision Tree

**FIGURE 1:**
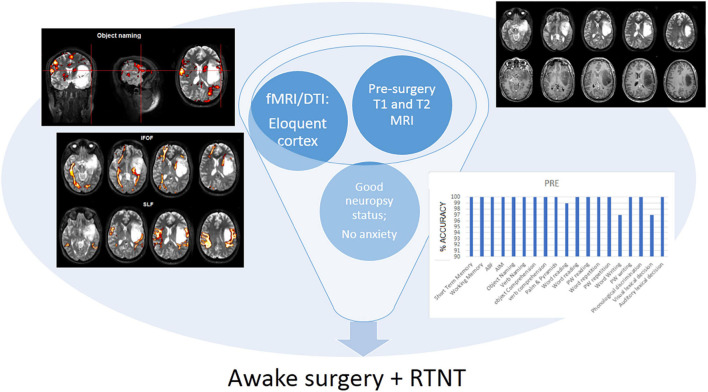
Decision tree for awake surgery plus RTNT procedure. In the case presented here, we found that the patient—male, young (25 year old), right-handed—had a low-grade glioma involving the temporo-insular cortex. The fMRI and DTI examination indicate that the brain tumor is infiltrating the functional tissue. The neuropsychological assessment showed that the cognitive performance of the patient is within the normal range on the majority of the tests administered, and provided the patient is not feeling particularly anxious/depressed, awake surgery plus RTNT were selected.

Following the initial neurosurgical consultation and MRI examination, patients with certain features (i.e., brain tumor involving the eloquent cortex) undergo fMRI and DTI along with presurgical neuropsychological assessment. If fMRI and DTI measurements indicate that the brain tumor is infiltrating or rearranging the functional tissue and the clinical characteristics of a patient and cognitive performance are within the normal range on the majority of the tests administered, and provided the patient is not feeling particularly anxious/depressed, awake surgery plus RTNT is discussed as an option (see Figure 1).

### Presurgery Neuropsychological Assessment

Extensive neuropsychological assessment was performed on the same day as fMRI and DTI examinations. Neuropsychological assessment covered different cognitive domains, and language skills were specifically tested if the brain tumor was localized in the left hemisphere.

The cognitive functions of a patient were tested by administering a neuropsychological battery involving general functioning as well as language processing (Table 1).

**TABLE 1:** Neuropsychological status of the patient: presurgery, immediately postsurgery (imm. post), and at follow-up.

**Cognitive domain**	**Function**	**Test**	**References**		**Pre**	**Imm. post**	**FU**
Fluid intelligence	Logic reasoning	Raven’s colored progressive matrices	Basso et al., 1987	RS	36	–	–
				CS	33,5	–	–
				ES	4	–	–
Executive functions	Strategy use	Verbal fluency	Novelli et al., 1996	RS	41	40	33
				CS	35	34	27
				ES	4	4	3
	Digit span	Backward test	Monaco et al., 2015	RS	5	6	4
				CS	4,42	5,42	3,42
				ES	4	4	2
Short term memory	Digit span	Forward test	Monaco et al., 2015	RS	7	6	7
				CS	6,44	5,44	6,44
				ES	4	3	4
Praxis	Upper limb imitation	Ideomotor apraxia	De Renzi et al., 1980	RS	72	72	72
				PE	4	4	4
	Buccofacial imitation	Oral apraxia	Spinnler and Tognoni, 1987	RS	20	20	20
				CS	19,75	19,75	19,75
				ES	4	4	4
Language	Comprehension	Token test	De Renzi and Faglioni, 1978	RS	36	36	36
				CS	33,5	33,5	33,5
				ES	4	4	4
		Objects comprehension	Miceli et al., 1994	Cut-off 39	40	40	40
		Verbs comprehension	Miceli et al., 1994	Cut off 19	20	20	20
	Semantics	Semantic fluency	Novelli et al., 1996	RS	44	56	50
				CS	38	50	44
				ES	3	4	4
		Object naming	Miceli et al., 1994	Cut-off 28	30	30	29
		Verb naming	Miceli et al., 1994	Cut off 26	28	27	26
	Semantic memory	Pyramids and palm trees test	Gamboz et al., 2009	RS	52	51	50
				CS, cut off 40,15	49,69	48,69	50,03
	Writing	Word and pseudoword writing	Luzzatti et al., 1994	Cut off 166,75	182	182	181
	Reading	Word reading	Ripamonti et al., 2014	Cut off 78	80	79	80
		Pseudoword reading	Ripamonti et al., 2014	Cut off 18	20	20	20
		Phrase reading	Miceli et al., 1994	Cut off 6	6	6	6
		Three-syllabic Word reading	Toraldo et al., 2006	Cut off 24	24	24	24
	Repetition	Word repetition	Miceli et al., 1994	Cut off 80	80	80	80
		Pseudoword repetition	Miceli et al., 1994	Cut off 20	20	20	20
		Sentence repetition	Miceli et al., 1994	Cut off 14	14	**13**	14
		Syntagm repetition	Miceli et al., 1994	Cut off 6	6	6	6
	Phonology	Phonological discrimination	Miceli et al., 1994	Cut off 58	60	60	60
	Lexical processing	Visual lexical decision	Ripamonti et al., 2016	Cut off 136	**142**	**136**	**137**
	Lexical processing	Auditory lexical decision	Ripamonti et al., 2016	Cut off 121	**126**	**126**	**125**

*RS, raw score; CS, corrected score; and ES, equivalent score.*

*Bold values denote pathological performances.*

Raw scores (RS) for each task were converted into correct scores (CS) for age, education, and sex and then converted into equivalent scores (ES). An ES of 0 indicates pathological performance, PE of 1 borderline performance, and a score of 2–4 indicates normal performance. The reference cutoff value was used for tests in which RS could not be converted into ESs (see Table 1).

### Presurgery Functional Magnetic Resonance Imaging and Diffusion Tensor Imaging

#### Functional Magnetic Resonance Imaging Tasks

Language fMRI tasks included object naming, verb naming, word and pseudoword reading aloud, and tongue movements. Tongue-movement localizer was performed to map the tongue motor-related areas involved in speech that could be taken as a reference for the planning and for the intra-surgery neuronavigation. As far as language mapping is concerned, multiple fMRI language related tasks were used to increase evidence and to obtain confirmation of the fMRI cluster more involved in/or close to the lesion.

In the silent object naming task, each block (*N* = 4, 15 s each) included seven trials (2,430 ms each). Stimuli were selected from the battery for the analysis of language disorders (Miceli et al., 1994). In the baseline condition (*N* = 5), a fixation cross (15 s) was presented between blocks.

In the silent verb naming task, each block (*N* = 4, 15 s each) included seven trials (2,430 ms each). Stimuli were selected from the battery for the analysis of language disorders (Miceli et al., 1994). In the baseline condition (*N* = 5), a fixation cross (15 s) was presented between blocks.

In the tongue localizer task, each block (*N* = 4, 15 s each) instructed the patient to make continuous circular tongue movements with closed mouth. In the baseline condition (*N* = 5), a fixation cross (15 s) was presented between blocks.

In the reading task, each block (*N* = 11, 15 s each) included 10 trials (1,500 ms each). Eight blocks included words, and three blocks pseudowords. Stimuli were selected from the battery for the analysis of language disorders (Miceli et al., 1994). In the baseline condition (*N* = 12), a fixation cross (15 s) was presented between blocks.

The Presentation^®^ software (Version 9.9, Neurobehavioral Systems Inc., CA, United States) was used for stimuli presentation and their synchronization with the MR scanner. Patients viewed the stimuli via VisuaStim Goggles (Resonance Technologies, Northrige, United States). Prior to acquisitions, participants practiced the tasks outside the scanner.

#### Functional Magnetic Resonance Imaging and Diffusion Tensor Imaging Data Acquisition and Analyses

The MRI was performed 6–10 days prior to craniotomy. A Philips Achieva 3-T (Best, Netherlands) whole-body scanner was used to acquire DTI, a SENSE-Head-8 channel head coil was used to acquire anatomical and functional images, and a custom-built head restrainer was used to minimize head movements.

Functional images were obtained using a single-shot gradient echo, echoplanar imaging (EPI) sequence. EPI volumes (*N* = 54) contained 34 axial slices (repetition time [TR] = 2,500 ms, echo time [TE] = 35 ms, field of view [FOV] = 230 mm, matrix: 128 × 128; slice thickness of 3 mm with no gaps, 90° flip angle, voxel size: 1.79 mm × 1.79 mm × 3.3 mm) and were preceded by four dummy images so that the MR scanner could reach a steady state.

Diffusion tensor data were acquired using an axial diffusion-weighted, single-shot, spin-echoplanar imaging sequence covering the whole brain (TR = 8,800 ms, TE = 74 ms, bandwidth = 1,287 Hz/pixel, flip angle = 90°, FOV = 224 cm × 224 cm; 70 contiguous axial slices, 1.6-mm slice thickness; matrix size = 224 × 224 voxels). Two *b* values were used; seven images at 0 s/mm^2^ (no diffusion weighting) and 64 non-coplanar images at 1,000 s/mm^2^ (diffusion weighting *b* value) were acquired. Gradient directions were uniformly distributed on a sphere.

High-resolution T2-weighted and post-gadolinium contrast T1-weighted anatomical MR images were acquired for the intra-operatory stereotactic surgical navigation system. To this purpose, a T1-weighted 3D magnetization-prepared, rapid acquisition gradient-echo fast field echo (T1W_3D_TFE SENSE) pulse sequence (TR = 8,200 ms, TE = 376 ms, FOV = 240,000 mm, 190 sagittal slices of 1-mm thickness, flip angle = 8°, voxel size: 1 mm × 1 mm × 1 mm) and a T3-weighted 3D magnetization-prepared, rapid acquisition gradient-echo fast field echo (T2W_3D_TFE SENSE) pulse sequence (TR = 2,500 ms, TE = 35 ms, FOV = 240,000 mm, 190 sagittal slices of 1-mm thickness, flip angle = 90°, voxel size: 1 mm × 1 mm × 1 mm) were acquired.

All calculations were performed on UNIX workstations (Ubuntu 8.04 LTS, i386, http://www.ubuntu.com/) using FSL (FMRIB Software Library version 6.0, Oxford, United Kingdom). Dummy images were discarded prior to further image processing. For fMRI data, standard preprocessing and single subject statistics were calculated. DTI data were analyzed using three-dimensional tract reconstructions from DTIStudio (Johns Hopkins, Baltimore, MD, United States) (version 3.0.3) software (Jiang et al., 2006).

### Awake Surgery

#### Intra-Surgery Real-Time Neuropsychological Testing Protocol for the Left Temporo-Insula Area

**FIGURE 2:**
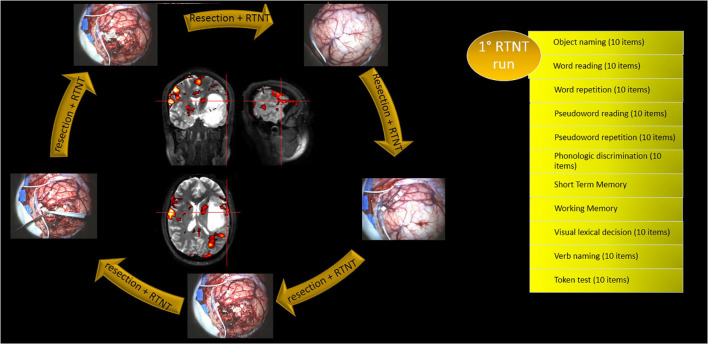
Alternation of DES mapping and RTNT together with the RTNT protocol for left temporal resections. The first run of tasks is reported as an example. Each task includes 10 items.

We administered the RTNT protocol for left temporal resections (see Figure 2). Importantly, the same tasks that were used during fMRI examination were also used during RTNT. Obviously, the fMRI examination has time constraints; therefore, tasks were selected according to lesion localization.

Some operational definitions are listed as follows:

• “RTNT runs” refers to a complete sequence of tests; once an RTNT run is completed, a second RTNT run is administered by the neuropsychologist, and so on, depending on the time needed for resection.
• “RTNT test” is a specific test, e.g., naming or reading. It appears once every RTNT run.
• “RTNT items” refers to the number of pictures, or words, or sentences (typically 10) included in each RTNT test.

Direct cortical stimulation mapping was performed at the beginning and repeated if needed (see Figure 2 and Table 2). Resection was then followed and was continuously paired with RTNT.

**TABLE 2:** RTNT performance in Case A.

	**1st Run**	**2nd Run**	**3rd Run**	**4th Run**	**5th Run**	**6th Run**
Object naming	100	100	100	100	90 (resting)	100
W reading	100	100	100	100	100	
W repetition	100	100	100	100		
PW reading	100	100	100	100	100 (feel asleep)	
PW repetition	100	80	70	Feel asleep, intelligible		
Phonological discrimination	93,75	73,33	68,75	86,66		
STM	100	100	60 (tiredness)			
WM	100	75				
Visual lexical decision	90	90	100	100		
Verb naming	90	90	100	90	100 (feel asleep)	
Token test	100	100	80	80		

### Immediate Postsurgery Assessment

The same neuropsychological evaluation performed presurgery was performed again 1 week after surgery.

### Follow-Up

Patients were routinely retested 4/5 months postsurgery. Patients perform the same neuropsychological tests and fMRI and DTI measurements as those administered presurgery.

## Results

### Presurgery Neuropsychological Assessment

Patient A is a 25-year-old right-handed man with 13 years of schooling. He is native Italian, with normal or corrected-to-normal vision; he has no history of psychiatric disease or drug abuse.

He reported partial seizures that occurred every day and lasted about 1 min. MRI revealed a LGG (which was confirmed by the postsurgery histological examination evidencing a glial neoplasm). The neuroradiological examination reported that the lesion involved the left fronto-temporo-insular cortex (hyperintense in T2-weighted MRI image, with no contrast-enhancing signs). During the neurosurgical consultation, a presurgery neuropsychological assessment was requested together with an fMRI and DTI examination.

As evidenced by the neuropsychological assessment, the neuropsychological status of the patient was good in almost every cognitive domain (see Table 1). He only performed below average on the visual and auditory lexical decision test.

Patient A was calm, collaborative, and did not feel anxious as revealed by the State-Trait Anxiety Inventory, a measure of trait and state anxiety (Spielberger, 1989).

### Presurgery Functional Magnetic Resonance Imaging and Diffusion Tensor Imaging Examination

**FIGURE 3:**
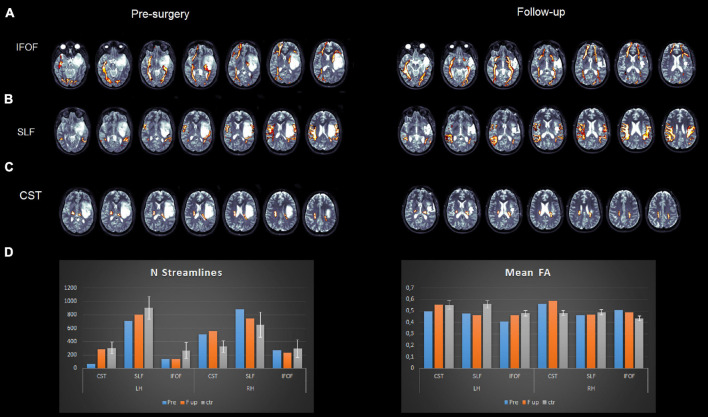
Pre- (left panel) and post (right panel)-surgery fMRI maps for tongue movements **(A)**, object naming **(B)**, verb naming **(C)**, and word/pseudoword reading **(D)** tasks. Activations are superimposed on the T2-weighted MRI axial slices of the patient.

The fMRI examination showed the following:

• The premotor part of the tongue representation is activated mainly contralesionally, at the level of the upper part of the lesion within the premotor cortex.
• Object naming-related activation is found in the left basal temporal area, posterior to, and not affected by, the lesion; activation in the lower premotor cortex is located anterior to the lesion. An additional fMRI cluster was found in the contralesional hemisphere.
• Verb naming-related activation showed similar results as compared to object naming. In addition, the left supramarginal gyrus activation is posterior to the lesion and not contiguous to it.
• Activation in Broca’s area related to reading was localized just in front of the lesion, and a part of the fMRI cluster was found medially. In addition, posterior temporal activation was found in the back of the lesion, bilaterally, and bilateral activation in the Rolandic operculum was found just in front of the lesion (see Figure 3).

**FIGURE 4:**
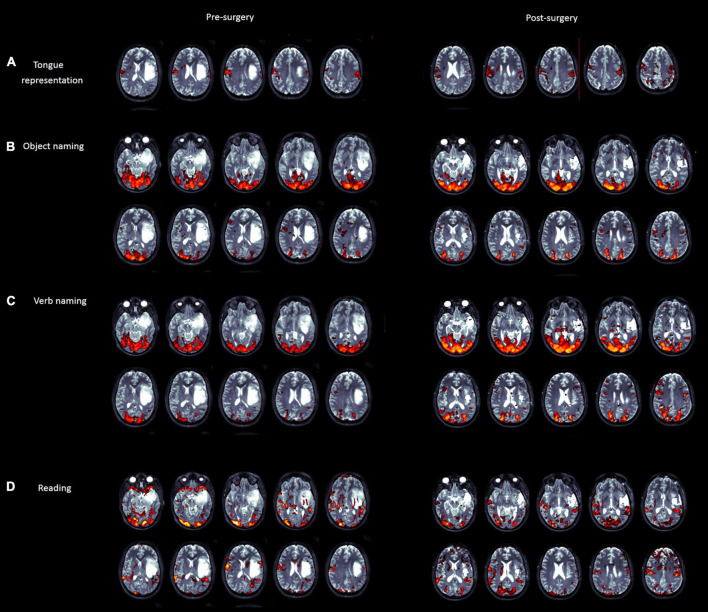
Pre- (left panel) and post (right panel)-surgery DTI reconstructions of the inferior fronto-occipital fasciculus **(A)**, superior longitudinal fasciculus **(B)**, and corticospinal tract **(C)**. Plots **(D)** represent mean fractional anisotropy (FA) and the number of streamlines for the patient and healthy controls.

DTI measurements showed the following:

• The inferior fronto-occipital fasciculus run medially to the lesion and the superior longitudinal fasciculus run externally to it (see Figure 4).
• The number of streamlines of the patient associated with each fascicle and the fractional anisotropy (FA) values were compared with our data of healthy controls (*N* = 25). We employed independent *t*-tests that were modified for small sample sizes, using SINGLIMS.EXE (Crawford and Garthwaite, 2002). SINGLIMS tests whether the score of an individual is significantly different from a control or normative sample and provides a point estimate of the abnormality of scores, i.e., it estimates the percentage of the population that would obtain a lower score. It then provides 95% confidence limits on this percentage. The analysis revealed that the FA of the patient of the left superior longitudinal fasciculus (SLF) and left inferior fronto-occpital fasciculus (IFOF) was significantly lower than the control group. In the right hemisphere, the FA of the patient for the corticospinal tract and IFOF was significantly higher than in controls. As to streamlines, we found that the number of streamlines of the patient for the left corticospinal tract was significantly lower than controls, while it was significantly higher for the right corticospinal tract (see Figure 4 and Table 3).

**TABLE 3:** Comparisons of the fractional anisotropy of the patient and number of streamlines vs. healthy controls (*N* = 25).

	**LH**			**RH**		
	**CST**	**SLF**	**IFOF**	**CST**	**SLF**	**IFOF**
**Fractional anisotropy**						
Pre	*t* = −1.572; *p* = 0.129	***t* = −2.399; *p* = 0.025**	***t* = −3.013; *P* = 0.006**	***t* = 3.898; *P* = 0.001**	*t* = −0.950; *P* = 0.351	***t* = 3.557; *P* = 0.002**
FU	*t* = 0.110; *P* = 0.913	***t* = −2.876; *P* = 0.008**	*t* = −0.825; *P* = 0.418	***t* = 5.277; *P* = 0.001**	*t* = −0.778; *P* = 0.444	***t* = 2.391; *P* = 0.025**
**Number of streamlines**						
Pre	***t* = −2.675; *P* = 0.013**	*t* = −1.168; *P* = 0.254	*t* = −1.052; *P* = 0.303	*t* = 2.068; *P* = 0.05	*t* = 1.243; *P* = 0.226	*t* = −0.177; *P* = 0.861
FU	*t* = −0.191; *P* = 0.85	*t* = −0.612; *P* = 0.546	*t* = −1.069; *P* = 0.296	***t* = 2.621; *P* = 0.015**	*t* = 0.663; *P* = 0.514	*t* = −0.435; *P* = 0.667

*Bold: significant differences.*

### Awake Surgery

Initial DES mapping was performed during a counting task and an object naming task, at 2.5 mA. Seven series of counting from 1 to 10 were administered. Negative mapping (i.e., DES did not elicit a response) was obtained. DES during object naming elicited motor speech arrest (i.e., with visible orbicularis oris muscle contraction) for three times.

Resection was then followed and was continuously paired with RTNT. Assessment went smoothly, with performance always on a high level (see Table 2). A slight decrement in accuracy was observed for pseudoword repetition, short-term memory, and working memory, for phonological processing and verbal comprehension (Token test). Object naming performed during DES confirmed the initial positive site reported above, where DES elicited a motor speech arrest. No other positive DES sites were found during white matter stimulation. Errors in pseudoword repetition consisted of consonant substitutions (*d*ilanciario in place of bilanciario, *f*roledario in place of froledano, and gu*f*ico in place of gupico), or regularizations (e.g., comando in place of coman*g*o and compito in place of *pontico*). At the phonological processing level, the patient matched a word with a wrong phonological target (e.g., maglia/shirt in place of vaglia/money order). The initial short-term memory span of Patient A was 6 and fell to 3 at the last RTNT run. Lexical-decision performance was considered stable since some errors made presurgery were also made during the RTNT. Finally, on the verbal comprehension task, the patient indicated the wrong token in response to the verbal request of the neuropsychologist twice out of 10 RTNT items. In the second half of the RTNT, the patient appeared tired at times and fell asleep. The patient completed four RTNT runs, where the object naming task was repeated two additional times and the word-reading task one additional time, and resection ended. A short break was made after the fifth repetition of object naming. We have previously reported (Skrap et al., 2016) that during the tumor removal, temporary interruption of the patient performance during the testing may occur. We defined this transitory decrement as a reversible neuropsychological deficit (Skrap et al., 2016). We speculated that oscillation in performance, with worsening and recovering accuracy in response within a few minutes. Due to the manipulation of the tissue, similar oscillation could be observed when some surgical traction is applied close to the corticospinal tract or in the medulla. In that case motor and somatosensorial evoked potentials may be temporarily altered.

Total resection was performed. Histological examination confirmed the presence of a glial neoplasm.

### Immediate Postsurgery Neuropsychological Examination

The immediate postsurgery performance of Patient A was within the normal range like his presurgery assessment. A non-ceiling performance on the lexical decision test, both on auditory and visual versions, was observed which was in keeping with his presurgery assessment.

### Neuropsychological Follow-Up

The performance of Patient A at the follow-up of 5 months after surgery was impaired only on auditory and visual lexical decision. All other tests were within the normal range. These data confirmed the result that there was no change in the performance of the patient pre-postsurgery and at follow-up. We can speculate that this result could be due to different factors. First, Plasticity could be a first reason. Indeed, the fMRI pre- and post-surgery maps show that a contra-lesion activation emerged postsurgery for language tasks. Second, intra-surgery neuropsychological monitoring through the RTNT, could early detect cognitive decrements and prevent deficit. Finally, the pre-surgical fMRI maps showed that the tissue within the hyperintense signal in T2- weighted MRI image was almost not activated, thus it might be not functional. Activations were mainly found at the anterior and posterior lesion margin, and these areas were exposed during craniotomy and were preserved.

### Functional Magnetic Resonance Imaging and Diffusion Tensor Imaging Follow-Up

The fMRI of Patient A follow-up examination revealed the following:

• Tongue movements elicited a similar activation map as presurgery, with bilateral distribution (presurgery activation was higher in the contra-lesion hemisphere).
• Object naming elicited an activation at the level of Broca’s area anterior to the resection cavity as compared to presurgery.
• Verb naming triggered a wider activation in the premotor cortex and supramarginal gyrus/parietal lobe.
• Reading elicited a similar activation map as presurgery.

The DTI analysis showed the following:

• All tracts were reconstructed normally, as in the presurgery analysis.
• The number of streamlines of the patient associated with each fascicle and the FA values were compared to our data of healthy controls (*N* = 25). The analysis revealed that FA of the left SLF was significantly lower than controls, while the FA of the right corticospinal tract and of the right IFOF was significantly higher than controls (Figure 4).

## Discussion

We presented the RTNT protocol for resections in the left temporal lobe and a case report. We chose to present a single case rather than our experience with the entire series of temporal patients in order to detail the entire RTNT protocol and performance, the procedure, fMRI images, and DTI results.

Our experience suggests that DES remains the gold standard in awake surgery; nonetheless, the surgeon can use the RTNT technique to increase the amount and type of available information about the cognitive status of the patient.

Studies investigating the postoperative cognitive status of patients often report deficits in functions that were not directly assessed during DES, since DES is mainly matched to object naming or counting tasks. In this specific patient, too, DES was performed during object naming and showed a positive speech arrest site. The RTNT added considerable information. This RTNT protocol for temporal resections includes tests that are most commonly used in awake surgery (e.g., see introduction mentioning naming, reading, repetition). In addition, it includes monitoring of short-term memory and working memory, phonological processing, lexical decision, and verbal comprehension (Token test) as well as verb naming.

The same tests were then assessed postsurgery. This is possible since RTNT takes advantage of resection time as the time of test presentation. In this way, surgery time is not extended; rather it is optimized, thanks to the online feedback provided to the surgeon, which enables him to proceed in a more confident manner, when RTNT demonstrates that the patient is responding proficiently to testing.

In the case report illustrated, our patient showed slight decrements in accuracy for pseudoword repetition, short-term memory and working memory, phonological processing, and verbal comprehension (Token test). His postsurgery testing performance was within the normal range. Based on our experience with RTNT, we believe that the lower performance on short-term memory and working memory may be due to fatigue, since immediate postsurgery performance was perfect. The patient was otherwise highly collaborative. In addition, his initial level of vigilance was high while in the final part of the RTNT he was tired and fell asleep. One disadvantage of the RTNT is that it presupposes levels of collaboration and flexibility, which can affect neuropsychological scores such as, in this case example, short-term memory and working memory. It is likely that the slight decrease in the Token test, too, was due to the decrease in short-term memory performance (Pisoni et al., 2019). The warning errors that we considered with much attention during surgery are phonological errors. The patient made some phonologic errors, both in production and in perception. Such errors occurred toward the end of resection.

Our multimodal approach combined RTNT, fMRI, and DTI together with this method. Combining this approach with DES presents some advantages in terms of extent of resection. In a previous study (Ius et al., 2019) using the multimodal approach, we reported a median extent of resection of 98%, compared with a median extent of resection of 83% with DES alone.

Preoperative mapping with fMRI and DTI provides essential, yet insufficient, information. In our opinion, it provides insight during surgery, but only neuropsychological testing can provide accurate levels of information about the cognitive status of the patient. The important aspect is the consistency between instruments and tests. Tongue movement, object naming, verb naming, word reading, and pseudoword reading were used in fMRI. The same tests were used intra-surgery. For example, a mistake made in the presurgery evaluation (e.g., patient saying “to marry” in response to a figure showing a shining ring shine instead of the verb “to shine”) and repeated during the intra-surgery examination as well is considered the baseline of the patient. The response will not be considered an error as it would happen in a formal neuropsychological assessment. In our example, some lexical decision errors were made both presurgery and during RTNT.

The postoperative assessment provides the information such as whether the patient activates or not the same areas as presurgically, whether performance on neuropsychological tests is the same as presurgically, as in our case report. Interestingly, the fMRI assessment also tells us if there is any plasticity or rearrangement pre- or postsurgery in glioma patients (Cargnelutti et al., 2020), showing, for example, that the postsurgery fMRI map for object naming shows an activation in Broca’s area that was not observed presurgery.

Postsurgery DTI assessment and fiber reconstruction indicates whether white matter pathways are preserved, and the results are interpreted in the light of the intra-surgery RTNT performance and according to which white matter pathway was detected during resection. In our case example, two data emerge. First, FA of the left IFOF was significantly lower pre-operatively compared with controls, while FA of the right IFOF was higher than controls both pre- and postoperatively, a sign of plasticity or pre-operative rearrangement. FA of the SLF was lower than controls presurgery and in the normal range postsurgery. Based on pre-operative data, follow-up values should never be viewed as absolute and isolated values but should be compared with pre-operative values. In addition, we are aware that we should be cautious in interpreting the DTI results above, as DTI measurements are sensitive to the presence of “free water” (Weninger et al., 2020). The presence of the tumor itself, or, postsurgery, the presence of the edema could temporarily modify the DTI measurements.

Taken together, the RTNT is highly informative, especially if combined with multimodality data. It does not require further effort. In neurosurgeries where DES is performed, the RTNT does not require additional materials or devices other than the same PC screen used to present DES stimuli. Finally, the RTNT provides essential information that can be used online, during surgery, for clinical aims to provide the surgeon with useful feedback. In addition, RTNT data can be used offline, for research purposes, by listening to patient performance recordings to detect error types and run error analysis or to correlate RTNT data with MRI data according to a multimodal approach.

## Data Availability Statement

The raw data supporting the conclusions of this article will be made available by the authors, without undue reservation.

## Ethics Statement

The studies involving human participants were reviewed and approved by CEUR fvg. The patients/participants provided their written informed consent to participate in this study.

## Author Contributions

MS and BT designed the research. BT, IG, MM, and TI performed the research. BT and MM analyzed the data. BT, MS, and IG wrote the manuscript. All authors edited the manuscript and revised the final version of the manuscript.

## Conflict of Interest

The authors declare that the research was conducted in the absence of any commercial or financial relationships that could be construed as a potential conflict of interest.

## Publisher’s Note

All claims expressed in this article are solely those of the authors and do not necessarily represent those of their affiliated organizations, or those of the publisher, the editors and the reviewers. Any product that may be evaluated in this article, or claim that may be made by its manufacturer, is not guaranteed or endorsed by the publisher.
